# In Vivo Characteristics of Premixed Calcium Phosphate Cements When Implanted in Subcutaneous Tissues and Periodontal Bone Defects

**DOI:** 10.6028/jres.115.021

**Published:** 2010-08-01

**Authors:** Akiyoshi Sugawara, Kenji Fujikawa, Satoshi Hirayama, Shozo Takagi, Laurence C. Chow

**Affiliations:** Sugawara Dental Clinic, and Nihon University School of Dentistry, Tokyo, Japan; Nihon University School of Dentistry, and Fujikawa Dental Office, Tokyo, Japan; Nihon University School of Dentistry, Matsudo, Japan; American Dental Association Foundation, Paffenbarger Research Center, National Institute of Standards and Technology, Gaithersburg, MD 20899, U.S.A

**Keywords:** animal study, histopathological examination, new bone formation, periodontal bone defects, premixed calcium phosphate cement

## Abstract

Previous studies showed that water-free, premixed calcium phosphate cements (Pre-CPCs) exhibited longer hardening times and lower strengths than conventional CPCs, but were stable in the package. The materials hardened only after being delivered to a wet environment and formed hydroxyapatite as the only product. Pre-CPCs also demonstrated good washout resistance and excellent biocompatibility when implanted in subcutaneous tissues in rats. The present study evaluated characteristics of Pre-CPCs when implanted in subcutaneous tissues (Study I) and used for repairing surgically created two-wall periodontal defects (Study II). Pre-CPC pastes were prepared by combining CPC powders that consisted of CPC-1: Ca_4_(PO_4_)_2_O and CaHPO_4_, CPC-2: α-Ca_3_(PO_4_)_2_ and CaCO_3_ or CPC-3: DCPA and Ca(OH)_2_ with a glycerol at powder-to-liquid mass ratios of 3.5, 2.5, and 2.5, respectively. In each cement mixture, the Ca to P molar ratio was 1.67. The glycerol contained Na_2_HPO_4_ (30 mass %) and hydroxypropyl methylcellulose (0.55 %) to accelerate cement hardening and improve washout resistance, respectively. In Study I, the test materials were implanted subcutaneously in rats. Four weeks after the operation, the animals were sacrificed and histopathological observations were performed. The results showed that all of the implanted materials exhibited very slight or negligible inflammatory reactions in tissues contacted with the implants. In Study II, the mandibular premolar teeth of mature beagle dogs were extracted. One month later, two-wall periodontal bone defects were surgically created adjacent to the teeth of the mandibular bone. The defects were filled with the Pre-CPC pastes and the flaps replaced in the preoperative position. The dogs were sacrificed at 1, 3 and 6 months after surgery and sections of filled defects resected. Results showed that one month after surgery, the implanted Pre-CPC-1 paste was partially replaced by bone and was converted to bone at 6 months. The pockets filled with Pre-CPC-2 were completely covered by newly formed bone in 1 month. The Pre-CPC-2 was partially replaced by trabecular bone in 1 month and was completely replaced by bone in 6 months. Examination of 1 month and 3 month samples indicated that Pre-CPC-2 resorbed and was replaced by bone more rapidly than Pre-CPC 1. Both Pre-CPC pastes were highly osteoconductive. When implanted in periodontal defects, Pre-CPC-2 was replaced by bone more rapidly than Pre-CPC-1.

## 1. Introduction

Previous studies have shown that calcium phosphate cement (CPC-1) [1–3], consisting of tetracalcium phosphate and dicalcium phosphate anhydrous, and CPC-2, consisting of α-tricalcium phosphate and calcium carbonate, mixed with sodium phosphate solution, were highly biocompatible [4–8] and osteoconductive [9–14]. Additionally enhancement of fast alkaline phosphatase (ALP-ase) activity [15] was essentially related to new bone formation and calcification. Water-free Pre-CPCs were also reported in previous studies. These were stable in the package, had good washout resistance, and only hardened after being exposed to a wet environment [16]. Former studies also reported that Pre-CPCs showed excellent biocompatibility [17–19]. Even though Pre-CPCs generally have longer hardening time and lower strength compared to the conventional CPC mixed with water, they have the following advantages: (1) ready to be used for clinical applications and (2) practically unlimited working time for the surgeon to apply the Pre-CPC to a desired site because it begins to harden and to form hydroxyapatite only after being exposed to water from surrounding tissues.

Besides uncertainty about how glycerol behaves in the body, there been always have questions whether the Pre-CPC could maintain its original shape in the body. Therefore, this study investigated *in vivo* characteristics of the Pre-CPCs when implanted in subcutaneous tissues in rats and used to repair surgically created two-wall periodontal defects in dogs.

## 2. Materials and Methods

### 2.1 Cement Powders

CPC-1 consisted of tetracalcium phosphate (TTCP:Ca_4_(PO_4_)_2_) (73 % mass fraction) and dicalcium phosphate anhydrous (DCPA:CaHPO_4_) (27 % mass fraction). CPC-2 consisted of α-tricalcium phosphate (α-TCP:α-Ca_3_(PO_4_)_2_) (90 % mass fraction) and CaCO_3_ (10 % mass fraction). CPC-3 consisted of DCPA (73 % mass fraction) and calcium hydroxide (Ca(OH)_2_) (27 % mass fraction). In each CPC powder, the Ca to P molar ratio was 1.67, the ratio found in stoichiometric hydroxyapatite (HA). TTCP was prepared by heating an equimolar mixture of commercially obtained DCPA (Baker Analytical Reagents, J. T. Baker Chemical Co., Phillipsburg, NJ)^1^ and CaCO_3_ (J. T. Baker Chemical Co.) at 1500 °C for 6 h in a furnace and quenched at room temperature. α-TCP was prepared by heating a mixture that contained 2 mol of DCPA and 1 mol of CaCO_3_ to 1500 °C for 6 h and then quenched in air. The powders were ground individually in a planetary ball mill in cyclohexane, or 95 % volume fraction ethanol, or without a liquid to obtain the desired median particle sizes based on data from previous studies. The median particle sizes of TTCP and DCPA for CPC-1 were 17 μm and 1 μm, respectively. The median particle sizes of α-TCP, CaCO_3_ and Ca(OH)_2_ for CPC-2 and -3 were (4 to5) μm, respectively. The particle sizes of powders were measured using a centrifugal particle size analyzer (SA-CP3, Shimadzu, Kyoto, Japan) with an estimated standard uncertainty of 0.2 μm.

### 2.2 Cement Liquid

A cement liquid containing glycerol (J. T. Baker Chemical Co.) (69.45 % mass fraction), hydroxypropyl methylcellulose (HMC) (Sigma Chemical Co., St. Louis, MO) (0.55 % mass fraction) and disodium phosphate (Na_2_HPO_4_) (Fisher Scientific Company, Fair Lawn, NJ) (30 % mass fraction) was prepared. HMC and Na_2_HPO_4_ served the functions of improving paste cohesiveness and accelerating cement hardening, respectively.

### 2.3 Preparation of Pre-CPCs

Pre-CPC-1, -2 and -3 were prepared by mixing CPC-1, 2 and 3 powder with the cement liquid at powder-to-liquid mass ratio (P/L ratio) of 3.5, 2.5 and 2.5, respectively. These ratios were chosen in order to produce pastes that exhibited workable consistencies.

### 2.4 Experimental Design of Study I (Biocompatibility)

This study was permitted by the Animal Experimentation Committee at Nihon University School of Dentistry, and performed in the animal and cell culture laboratories at the Nihon University School of Dentistry. The experiments followed the “Guidelines for Animal Experimentation Committee at Nihon University School of Dentistry.”

**Fig. 1:**
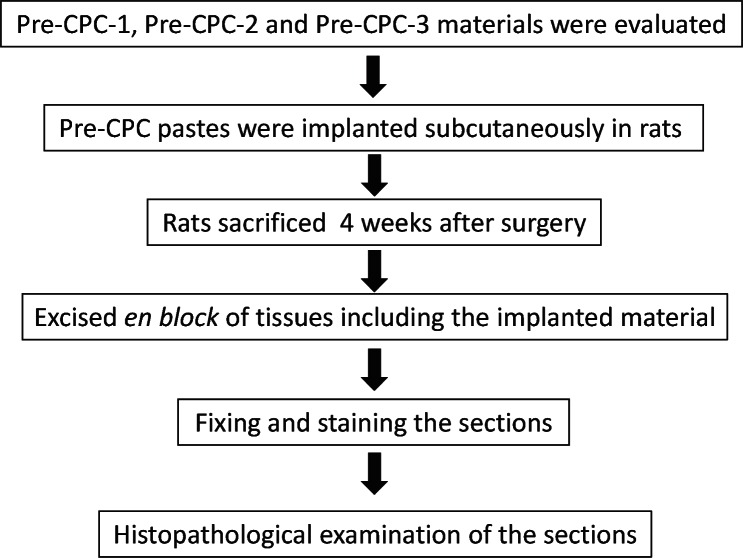
Experimental procedures of Study I.

**Fig. 2:**
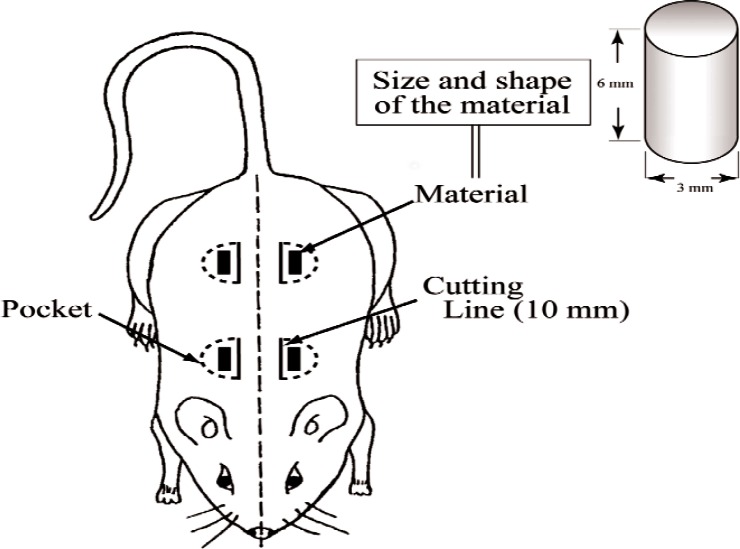
Scheme of the pocket created in back area.

**Fig. 3:**
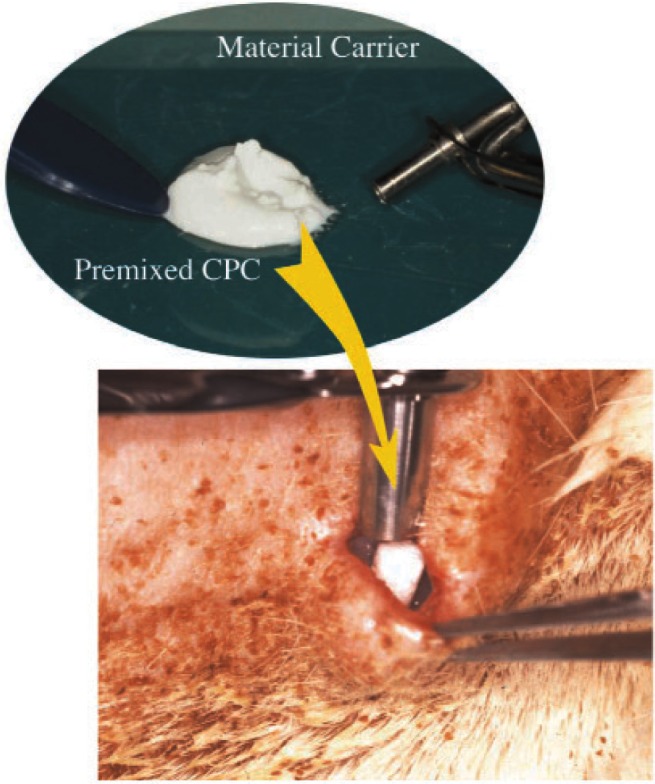
Implanted procedures of this experiment.

Experimental procedures of the study are shown in Fig. 1. Each experimental material was tested in five adult Donryu rats with an average body weight of 200 g to 250 g. All experimental procedures on given animals were completed as aseptic as possible. Each animal was anesthetized with pentobarbital sodium (Nembutal, Abbott Laboratories, North Chicago, IL, U.S/A.) injection at a dose of 1.5 mg/kg body mass. Under the general anesthesia, approximately 3 cm × 2 cm of back area of the rat was shaved and swabbed with 70 % volume fraction ethanol (Wako Pure Chemical Industries Ltd., Osaka, Japan). A 15 mm horizontal incision was made along the side of a back bone, and a skin pocket created 20 mm to 25 mm away from the back bone by blunt dissection on the subcutaneous lesion (Fig. 2). A total of four pockets were formed totally on each rat, and each pocket was separated a distance of more than 40 mm. Each cylinder shaped sample (3 mm diameter and 4 mm length) from the premixed CPC, was inserted into a pocket of subcutaneous tissues as shown in Fig. 3, and then the pocket was closed with interrupted sutures. The material sample was not hardened yet when the sample was inserted into the pocket. Four weeks after surgery, the animals were sacrificed and the tissues including the test materials were excised *en block*. Tissues were fixed in 10 % volume fraction neutralized-buffered formalin, decalcified with Plank-Rychlo solution and embedded in paraffin. Subsequently, paraffin embedded blocks of decalcified samples were cut into 6 μm sections, and stained with hematoxylin and eosin. Histopathological features of each section were observed using an optical microscope (Vanox-S, Olympus, Tokyo, Japan).

### 2.5 Experimental Design of Study II (Osteoconductivity)

Results from the subcutaneous tissue reaction (Study I) exhibited that each Pre-CPC showed negligible inflammatory reaction. The implanted material maintained original graft shape and was encapsulated with extremely thin fibrous connective tissues. Our previous study showed that CPC-1 and -2, mixed with a 0.5 mol/L sodium phosphate showed excellent biocompatibilities and osteoconductivities. Therefore, Pre-CPC-1 and -2 were selected as the experimental materials in this study.

**Fig. 4:**
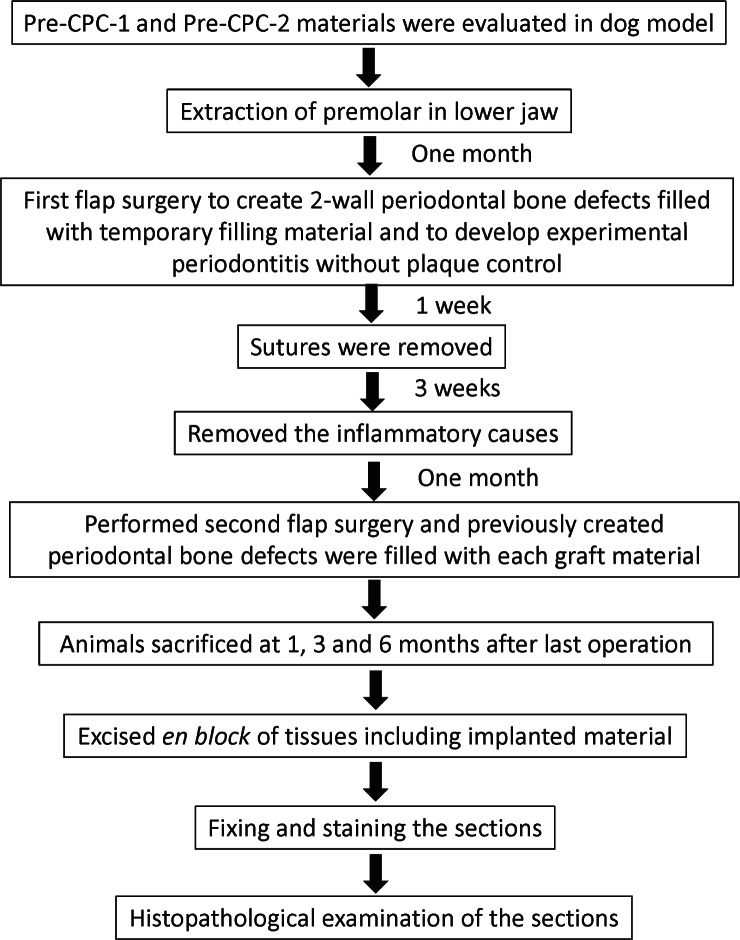
Experimental procedures of Study II.

The study protocol was reviewed and approved by the Animal Experimentation Committee at Nihon University School of Dentistry at Matsudo, and performed in the animal laboratory at Nihon University School of Dentistry at Matsudo. Mature (2 to 4 years old) beagle dogs were used in this study. The outline of the study is illustrated in Fig. 4.

The investigation of each experimental material was carried out in three adult beagle dogs (average body weights were approximately 8 kg to 12 kg). Before starting this study, both left and right mandibular fourth premolar teeth were extracted to make enough space for the bone graft. All experimental procedures on each animal were completed without any interruption. The surgical procedures were performed under strict aseptic conditions. General anesthesia for each dog was administrated by intravenous injection of pentobarbital sodium (Nembutal sodium solution: Abbot Lab., North Chicago IL, U.S.A.; 0.5 ml/kg of body weight).

**Fig. 5:**
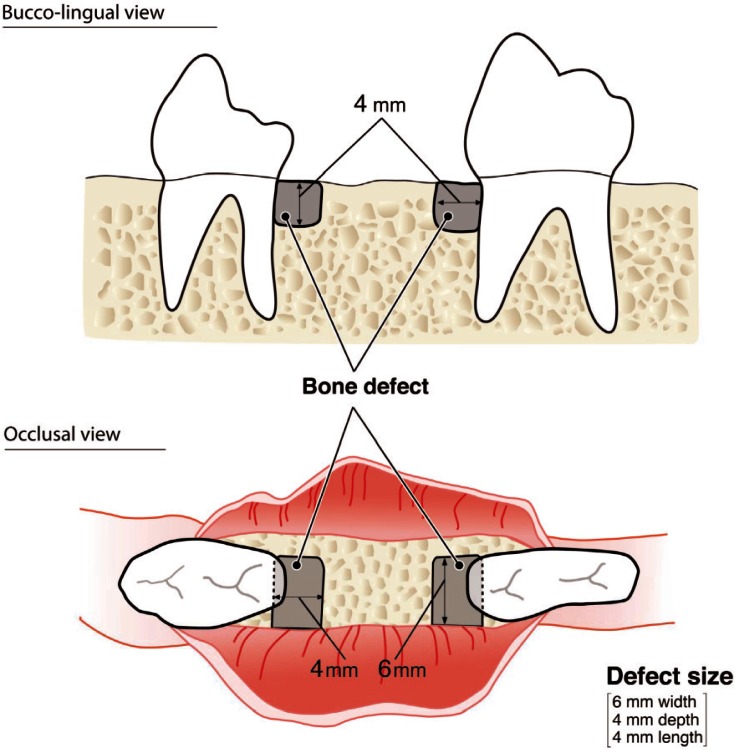
Scheme of surgically created bone defects.

One month later, surgical procedures were performed on the designated teeth under general anesthesia supplemented by local administration of lidocaine-HCL (2 % mass fraction Xylocaine, Astra Japan Ltd., Fujisawa Pharmaceutical Co., Ltd., Osaka, Japan) to reduce hemorrhage in the surgical site. Intracrevicular incisions were made on the facial and both mesial and distal interproximal surfaces. Full thickness envelope flaps were then reflected on the facial and interproximal surfaces extended apically just past the mucogingival junction. Two wall bone defects (4 mm width and depth) were created in the alveolar bone close to the mesial of the first molar, distal of the third premolar, mesial of the third premolar or distal of second premolar (Fig. 5). Each bone defect was filled with temporary filling material (Caviton: G-C Corp., Tokyo, Japan) to the level of the neighboring bone to maintain the surgically created bony defect and acts as a source of inflammation. The flaps were replaced in their preoperative positions and secured with interrupted 4-0 silk sutures (Ethicon Inc., NJ, U.S.A.). All dogs were fed a soft diet without any plaque control to achieve the artificially created periodontal disease conditions.

**Fig. 6:**
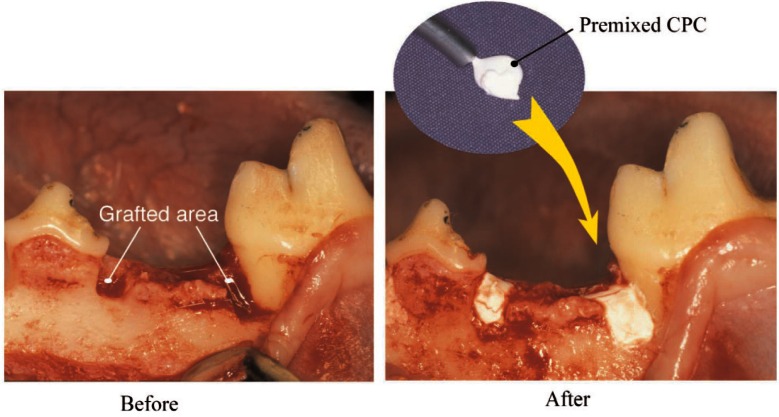
Grafting procedures of this experiment.

Sutures were removed one week after the surgery. The temporary filling material was removed 4 weeks later. After an additional four weeks, a second surgical procedure was performed. Before surgery, clinical measurements (free gingival margin to the base of the pocket and free gingival margin to the cementoenamel junction) and gingival index (scores of inflammatory [20]) were recorded on the experimental teeth. A mucoperiosteal flap was elevated after an incision in the gingival sulcus except in interproximal areas, where incisions were extended on the lingual line angle of each tooth to permit papillary reflection and not made superior on the defect site. Full thickness envelope flaps were then reflected on the facial and interproximal surfaces just past the mucogingival junction. Scaling and root planning were performed on the cervical area of the alveolar bone. The distance from cementoenamel junction to the crest of the alveolar bone and the bottom border of the bone defects was recorded in the mid interproximal area of each experimental root. Each bone defect was randomly assigned as either an experimental or control site. Experimental sites were treated with each Pre-CPC to fill the bony defect prior to flap closure (Fig. 6). The flaps were replaced at their preoperative position and sutured to secure complete coverage of the alveolar bone. Periodontal dressing and antibiotic medications were not provided. After surgery, daily plaque control was carried out using cotton balls moistened with saline. Two weeks after surgery, daily oral hygiene procedures were started using a soft nylon toothbrush moistened with 0.2 % chlorhexidine (Sigma Chemical Co. St. Louis, MO, U.S.A.). All dogs received a prophylaxis with fluoride phosphate prophylaxis paste (Prophy paste, Clean Chemical Sweden) once a week. The periodontal status was recorded as previously described for all three dogs at 1, 3 and 6 months after surgery.

#### 2.5.1 Histological Preparation of the Specimens

**Fig. 7:**
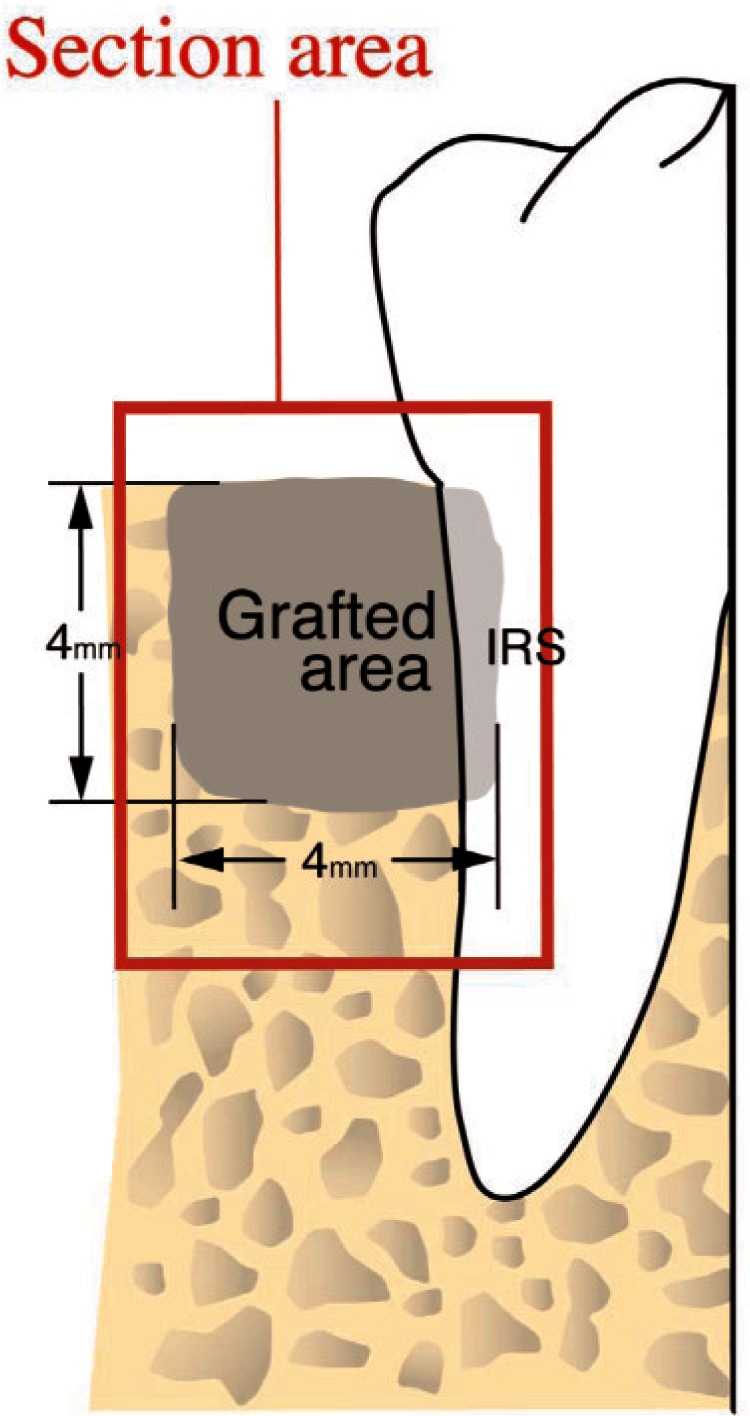
Sectioned view of grafted area.

One, 3 and 6 months after the operation, the animals were sacrificed and the tissues including the test materials were excised *en bloc*. The samples were fixed in 10 % neutralized buffered formalin, decalcified by Plank-Rychlo solution (Fujisawa Phamaceutical Co., Ltd., Osaka, Japan) and embedded in paraffin. Subsequently, paraffin-embedded blocks were mounted in a mesio-distal plane, serially sectioned at thickness of 7 μm thick and stained with hematoxylin and eosin. The section area in this experiment is shown in Fig. 7. Histopathological features of each section were observed using an optical microscope (Vanox-S, Olympus, Tokyo, Japan).

## 3. Results

All acronyms used in animal studies were shown in Table 1.

### 3.1 Study I (Fig. 8)

Pre-CPC-1: The grafted material (GM) was surrounded by thin fibrous connective tissues (FCT) with a quite few inflammatory cells. Small numbers of foreign body giant cells were found adjacent to the material. Tissue reaction to the material was extremely mild.

Pre-CPC-2: Relatively thin FCT surrounded the material. Few inflammatory cells were observed around the materials. Infiltrated connective tissues (ICT) were predominantly seen in mass of the material. Tissue reaction to the material was very mild.

Pre-CPC-3: The material was surrounded by thin FCT and small numbers of inflammatory cells. Multinuclear giant cells (MGC) were found in the thin connective tissues. Accumulated granulation tissue (GT) with fibrous cells was observed around the CPC mass. The histopathological reactions to this material were basically similar to those of CPC-1 and -2.

All pre-CPC implants were found to retain their original cylindrical shape despite that during the surgery the CPC in the pocket was not yet hardened at the time of suturing. In general, all Pre-CPCs showed similar histopathological reactions. No differences were observed among the pastes prepared with different CPC powders. Each experimental material was encapsulated by thin FCT with small numbers of foreign body giant cells. Some sections showed a few infiltrated cells adjacent to the material. Tissue reactions to all Pre-CPCs were very mild.

### 3.2 Study II (Fig. 9–11)

#### 3.2.1 Clinical Findings

All dogs demonstrated various degrees of gingival inflammation at the 2nd flap surgery. One week prior to being sacrificed, they showed a significant improvement of the gingival index. Before sacrifice, all dogs showed healthy periodontal condition without any gingival recession or inflammations. There were no significant differences between the control and the experimental surgical sites in clinical observations.

#### 3.2.2 Histological Findings at One Month After Surgery (Fig. 9)

Pre-CPC-1: The grafted area (GA) was covered by relatively thick FCT. The grafted material (GM), which should have mostly converted to HA, was partially replaced by newly formed bone (NB) without any inflammatory reaction. Multinuclear giant cells (MGC), similar in appearance to osteoclasts, appeared around the resorbing front where the material resorbed and was replaced by NB. Junctional epithelium (JE) attachment was prevented at the crestal level of instrumented root surface (IRS). Newly formed cementum (NC) was found on the apical side of IRS.

Pre-CPC-2: No inflammatory reactions were observed in the GA. Woven bone (WB) was formed throughout the entire GA and some trabecular bone (TB) was also noted. NC formation was observed around the apical area of IRS, and apical proliferation of JE was prevented at the crestal level of IRS. Bone formation in Pre-CPC-2 GA was slightly faster than that in Pre-CPC-1 GA.

#### 3.2.3 Histological Findings at Three Months After Surgery (Fig. 10)

Pre-CPC-1: No inflammatory reaction was observed in the GA. Most of the GM was converted to NB. The GA was completely covered with thin mature bone tissue with FCT. TB was formed throughout the GA, but clusters of GM were still present. NC was clearly formed and the JE proliferation was completely prevented at the crestal level of IRS. The number of phagocytic cells was reduced compared with the 1-month histological features of Pre-CPC-1.

Pre-CPC-2: The GA was covered by relatively mature bone tissues with dense FCT. Some GM remained in the GA, but the GA was mostly replaced by relatively mature TB with Harversian lamellae (HL). NC was generated along entire IRS. Phagocytic response was decreased compared with the 1-month sample of Pre-CPC-2.

#### 3.2.4 Histological Findings at Six Months After Surgery (Fig. 11)

Pre-CPC-1: The GM was completely resorbed and also converted to normal alveolar bone (AB). New bone was formed at the crest of AB with HL and osteocyte (OC). Osteoblastic activity was almost quiescent in the GA. Bone marrow (BM) was formed among TB. NC was found along on the entire IRS. A thin FCT layer, of which structure was closely similar to that of a periodontal ligament-like structure (PLS), was generated between AB and NC.

Pre-CPC-2: The defect was completely converted to normal AB with BM and HL and was covered with periosteum (PO) attached to FCT. NC was generated along entire IRS, and a PLS was clearly formed between AB and NC. Histological features were quite similar to those of Pre-CPC-1 at 6-months.

In general, both Pre-CPCs retained shape-integrity and restored the original alveolar bone shape after grafting of two-wall periodontal bone defects. Defects filled with the Pre-CPCs were replaced by natural bone within 6 months after the surgery. Periodontal tissues including NC and a PLS were also gradually regenerated. The defect filled with Pre-CPC-2 showed relatively faster bone replacement when compared to Pre-CPC-1.

## 4. Discussion

Reconstructive surgery for alveolar bone deficiencies, especially periodontal bone defects, can be performed with a number of techniques including guided bone regeneration, in which an occlusive barrier membrane is replaced between the connective tissues and residual alveolar bone to create a space for the new bone formation. However, it would be quite difficult to repair large defects, such as 1- or 2-wall periodontal bone defects with either membrane alone or in combination with bone grafting materials including autogenous bone. These grafting materials do not have sufficient properties, such as hardening, washout resistance, and bioresorption in the biological environment [16–19]. Therefore, it was difficult to surgically reconstruct alveolar bone defects for long time.

CPC and several other similar CPCs harden in 10 min with use of a phosphate solution as the liquid and form a resorbable HA crystalline scafold as the final product [16]. On the other hand, CPCs have some difficulty maintaining the original grafted shape at defect sights when used as implanted materials, because they do not have enough washout resistance and viscosity in the body fluid within their hardening periods [17–19]. Despite these properties, our previous studies still reported that alveolar ridge augmentation and 3-wall periodontal bone defect reconstructed by using CPC, without using a barrier membrane, were replaced by natural bone within 6 months after surgery [10,14]. Pre-CPCs are stable in the package and have sufficient viscosity and excellent washout resistance in body fluids, so that they would harden only after delivery to defect sites where glycerol-tissue fluid exchange occurs and could be prepared in advance under well-controlled conditions. An important handling property for the Pre-CPC is an adequate working time for the surgeon to place and shape the cement into the defect. Therefore, we assume that the Pre-CPC could be used to repair a variety of bone defects.

The results obtained from this study showed that the defect filled with either Pre-CPC-1 or 2 was gradually replaced by newly formed bone. The defect filled with Pre-CPC-2 showed singificantly faster bone formation than that filled with Pre-CPC-1. Our former study indicated that the alkaline phosphatase (ALP-ase) activity, which was closely related to new bone formation, was enhanced significantly under the presence of either CPC-1 or CPC-2 [21–23]. The activity of CPC-2 showed faster enhancement than that of CPC-1. In addition to those properties, CPC-2 contained carbonate in the original structures, so the final product of CPC-2 contained more carbonate apatite and had lower crystallinity than that of CPC-1 [1,24–26]. This poorly crystalline HA containing carbonate was easily absorbed by osteoclasts, so it might be expected that new bone formation occurred in the absorption site simultaneously. Therefore, as the results of the above properties, Pre-CPC-1 and -2 showed excellent osteoconductivity, especially showing faster bone formation occurred in the presence of CPC-2.

Six months after the surgery, the defects filled with either Pre-CPC-1 or -2 were converted to natural bone without using any barrier membrane. Reconstruction area of the Pre-CPC kept the original shape as when the material was originally implanted. Those results suggested that Pre-CPCs were not only useful as bone reconstructive materials, but also effective for any type of bone deficiencies.

## 5. Conclusion

Based on the results obtained from study I, all Pre-CPCs were shown to be highly biocompatible and retained the original cylindrical shape in subcutaneous tissues, thus suggesting that those materials may be useful for bone graft applications.

Pre-CPCs, when filled into artificially created two-wall periodontal bone defects, were resorbed and converted to natural alveolar bone within 6 months after surgery. One and 3 month results indicated that Pre-CPC-2 was resorbed and replaced by NB significantly faster than Pre-CPC-1. The faster implant-to-bone turnover may be attributed to lower crystallinity and higher carbonate content of the HA formed in Pre-CPC-2.

These results indicated that Pre-CPC-1 and -2 should be an effective and suitable material for large periodontal bone defects. Moreover, accelerated bone formation could be expected with Pre-CPC-2 when it is used as bone graft material.

## Figures and Tables

**Fig. 8:**
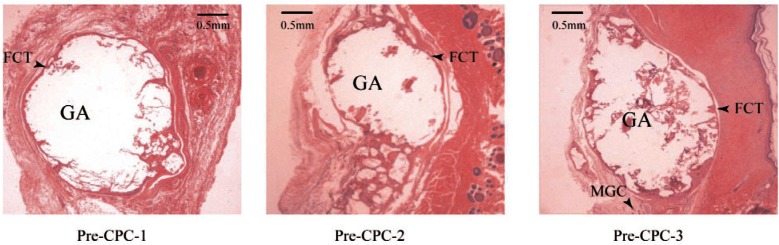
Study I—Four weeks after surgery. All Pre-CPCs showed quite similar histopathological reactions. The grafted material was encapsulated by thin fibrous connective tissues (FCT) with small numbers of infiltrated cells. Tissue reactions to all Pre-CPCs were very mild. All Pre-CPCs retained their original graft shapes.

**Fig. 9:**
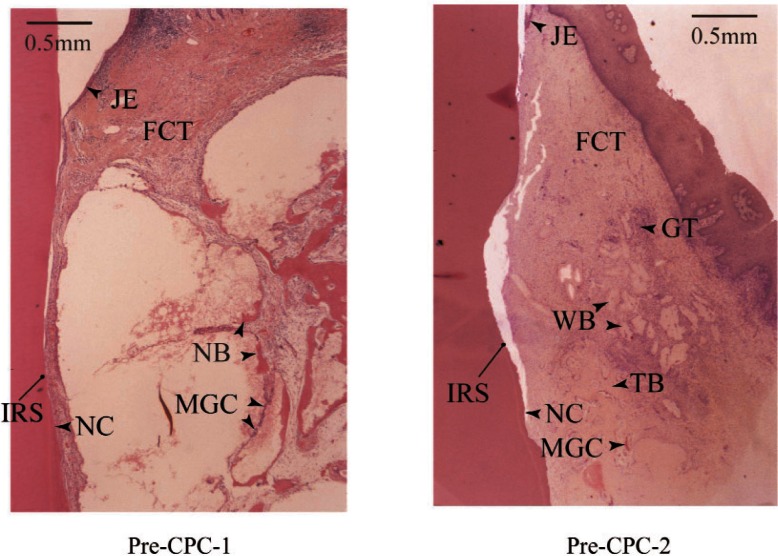
Study II—One month after surgery. Pre-CPC-1: Newly formed bone (NB) was formed partially in grafted area (GA). Junctional epithelium (JE) extention was prevented at the crestal level of instrumented root surface (IRS). Newly formed cementum (NC) was formed in apical side of IRS, Multinuclear giant cells (MGC), which resembled to osteoclasts, appeared around the grafted materials (GM). Pre-CPC-2: Woven bone (WB) was formed in entire GA. Trabecular bone (TB) was already formed partially. NC was observed around the apical area of IRS, and JE proliferation was prevented at the crestal level of IRS.

**Fig. 10:**
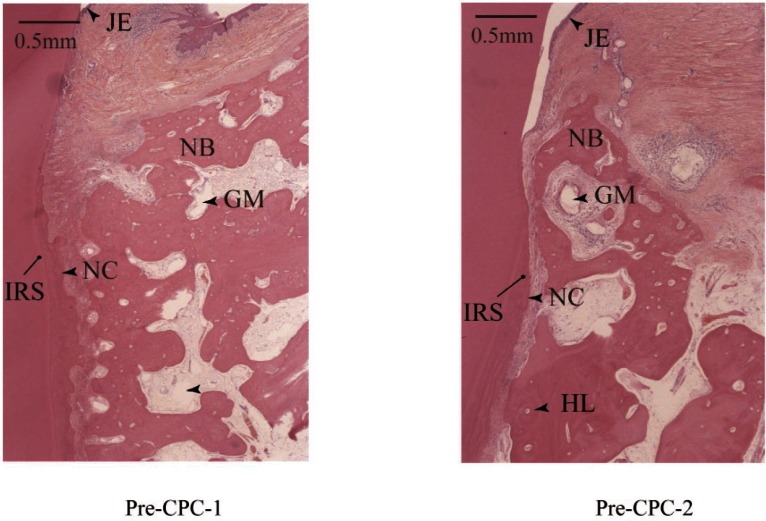
Study II—Three months after surgery. Pre-CPC-1 Most of GM was converted to NB, but GM clusters was still slightly present in GA. TB was formed throughout GA. NC was clearly formed and JE proliferation was prevented at crestal level of IRS. Pre-CPC-2: GA was mostly replace by nomal bone with Herversian lamellae (HL). NC was generated along entire IRS.

**Fig. 11:**
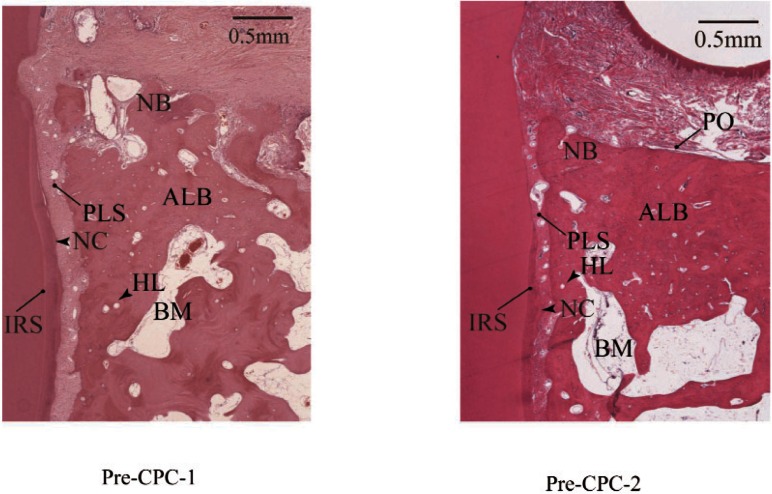
Study II— Six months after surgery. Pre-CPC-1: GM was completely converted to alveolar bone (ALB) with HL and osteocyte (OC). Bone marrows (BM) were formed among TB. NC was formed on entire IRS. Periodontal ligament-like structure (PLS) was generated between AB and NC. Pre-CPC-2: GA was replaced by natural AB with BM and HL, and was covered by periosteum (PO) attached to FCT. NC was generated along entire IRS. PLS was clearly formed between AB and NC.

**Table 1:** Acronyms used in Study I and Study II

Alveolar bone	AB	Junctional epithelium	JE
Bone marrow	BM	Multinuclear giant cells	MGC
Fibrous connective tissue	FCT	Newly formed bone	NB
Grafted area	GA	Newly formed cementum	NC
Grafted material	GM	Osteocyte	OC
Granulation tissue	GT	Periodontal ligament-like structure	PLS
Harversian lamellae	HL	Periosteum	PO
Infiltrated connective tissues	ICT	Trabecular bone	TB
Instrumented root surface	IRS	Woven bone	WB

## Footnotes

1 Certain commercial materials and equipment are identified in this paper to specify the experimental procedure. In no instance does such identification imply recommendation or endorsement by the National Institutes of Health or American Dental Association Foundation or National Institute of Standards and Technology or Nihon University or that the material or equipment identified is necessarily the best available for the purpose.
